# Efficacy of direct-to-operating room trauma resuscitation: a systematic review

**DOI:** 10.1186/s13017-023-00532-5

**Published:** 2024-01-18

**Authors:** Dongmin Seo, Inhae Heo, Donghwan Choi, Kyoungwon Jung, Hohyung Jung

**Affiliations:** 1 Division of Trauma Surgery, Department of Surgery, Ajou University School of Medicine, 164 Worldcup-Ro, Yeongtong-Gu, Suwon-si, Gyeonggi-do 16499 Republic of Korea; 2 Regional Trauma Center of Southern Gyeong-Gi Province, Ajou University School of Medicine, Suwon, Republic of Korea

**Keywords:** Direct-to-operating room, Trauma, Mortality, Treatment outcome

## Abstract

**Background:**

Hemorrhage control is a time-critical task, and recent studies have demonstrated that a shorter time to definitive care is positively associated with patient survival and functional outcomes. The concept of direct transport to the operating room was proposed in the 1960s to reduce treatment time. Some trauma centers have developed protocols for direct-to-operating room resuscitation (DOR) programs. Moreover, few studies have reported the clinical outcomes of DOR in patients with trauma; however, their clinical effect in improving the efficiency and quality of care remains unclear. In this systematic review, we aimed to consolidate all published studies reporting the effect of DOR on severe trauma and evaluate its utility.

**Methods:**

The PubMed, EMBASE, and Cochrane databases were searched from inception to April 2023, to identify all articles published in English that reported the effect of direct-to-operating room trauma resuscitation for severe trauma. The articles were reviewed as references of interest.

**Results:**

We reviewed six studies reporting the clinical effect of operating room trauma resuscitation. A total of 3232 patients were identified. Five studies compared the actual mortality with the predicted mortality using the trauma score and injury severity score, while one study compared mortality using propensity matching. Four studies reported that the actual survival rate for overall injuries was better than the predicted survival rate, whereas two studies reported no difference. Some studies performed subgroup analyses. Two studies showed that the survival rate for penetrating injuries was better than the predicted survival rate, and one showed that the survival rate for blunt injuries was better than the predicted survival rate. Five studies reported the time to surgical intervention, which was within 30 min. Two studies time-compared surgical intervention, which was shorter in patients who underwent DOR.

**Conclusion:**

Implementing DOR is likely to have a beneficial effect on mortality and can facilitate rapid intervention in patients with severe shock. Future studies, possibly clinical trials, are needed to ensure a proper comparison of the efficiency.

**Supplementary Information:**

The online version contains supplementary material available at 10.1186/s13017-023-00532-5.

## Background

Hemorrhage control is a time-critical task, and a recent study has shown that delays in bleeding control in patients with significant abdominal injuries were associated with a 1% higher mortality risk for each 3 min of delay in the operating room (OR) [1]. Moreover, the concept of the “golden hour” in trauma control emphasizes the significance of the time to intervention as a critical factor that improves the chances of survival of severely injured patients [2]. A recent study has demonstrated that a shorter time to definitive care is positively associated with patient survival and functional outcomes [3].

The concept of direct transport to the OR was proposed in the 1960s to minimize the treatment time for patients with trauma [4]. While some trauma centers have established protocols for direct-to-operating room resuscitation (DOR) programs, few studies have reported the clinical outcomes of DOR in patients with trauma, particularly those with penetrating injuries [5–11].

A recent systematic review of hybrid operating theaters (OTs) highlighted their ability to facilitate simultaneous interventional radiology and operative procedures for the treatment of severely injured patients; however, the cost–benefit ratio was unclear [12]. Moreover, the practice of DOR has not been systematically reviewed in the literature for decades. Therefore, this systematic review aimed to consolidate all published studies reporting the impact of DOR on severe trauma and evaluate its utility.

## Methods

This systematic review was conducted in accordance with the protocol registered in PROSPERO [http://www.crd.york.ac.uk/PROSPERO/] (reference number: CRD42023414650).

### Definition of DOR

DOR refers to the policy of transporting critically injured patients directly to the OR for resuscitation, bypassing the resuscitation suite [emergency department (ED) or trauma bay]. The decision to perform DOR is based on the discretion of the trauma team members based on the prehospital injury pattern and physiology of the patient.

### Search strategy

The PubMed, EMBASE, and Cochrane Library databases were systematically searched from inception to April 2023. The key search terms included “trauma,” “direct-to-operation room,” “mortality,” and “treatment outcome.” The complete search strategy is outlined in Additional file 1. The inclusion criteria were (1) English language studies, (2) full-text articles, and (3) studies that utilized the DOR protocol and evaluated the impact of direct-to-operating room trauma resuscitation in patients with trauma. The exclusion criteria were as follows: (1) gray literature and (2) abstracts, letters, editorials, expert opinions, technical notes, case reports, and reviews.

### Screening process and data extraction

All articles identified using the search strategy were independently screened by two investigators. The full texts were reviewed and assessed for eligibility based on the inclusion and exclusion criteria. Disagreements were resolved independently by a third reviewer at all stages. Data were extracted through discussions with a third reviewer. Data obtained from full-text articles included the year of publication, number of patients, study design, and inclusion and exclusion criteria. The outcomes of this systematic review included mortality and time to surgical intervention.

### Quality assessment and analysis

The quality of the observational studies was assessed using the Newcastle–Ottawa Scale. A detailed assessment of the risk of bias is presented in Table 1. The data analysis was qualitative, allowing overall interpretation of the data based on a qualitative summary. The results were reported in accordance with the PRISMA guidelines.

**Table 1:** Data regarding the Newcastle–Ottawa Scale for assessing the quality of the non-randomized studies

Study	Selection				Comparability		Outcome			Total score
	Is the case definition adequate	Representativeness of the cases	Selection of controls	Definition of controls	On the main factor	On other risk factors	Assessment of exposure	Same method of ascertainment for cases and controls	Non-response rate	
Wieck et al. [5]	*	*	–	*	*	*	*	*	–	7/9
Steele et al. [6]	*	*	–	*	*	–	*	*	–	6/9
Rhodes et al. [7]	*	*	–	*	*	–	*	*	–	6/9
Martin et al. [8]	*	*	–	*	*	–	*	*	–	6/9
Johnson et al. [9]	*	*	–	*	*	*	*	*	–	7/9
Habarth-Morales et al. [10]	*	*	–	*	*	*	*	*	–	7/9

## Results

### Included studies

**Fig. 1:**
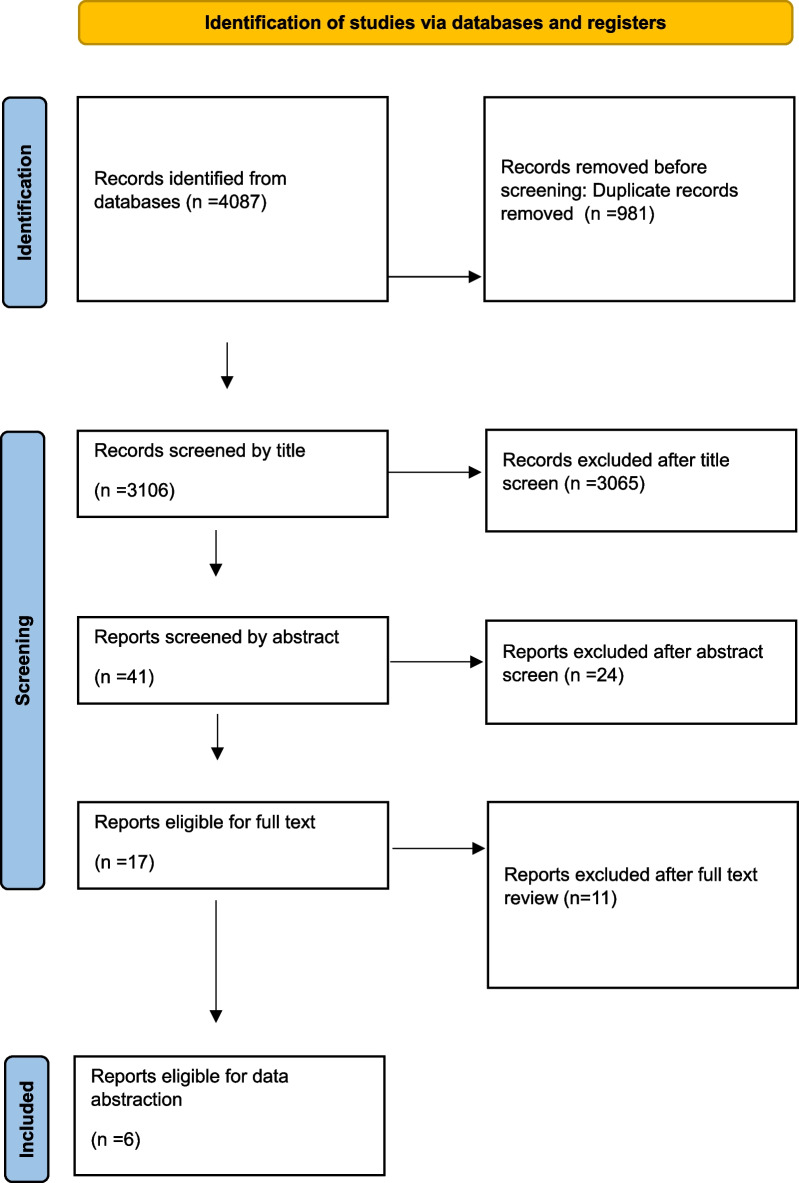
PRISMA flow diagram regarding the literature search. *PRISMA* preferred reporting items for systematic reviews and meta-analyses

The literature search yielded 4087 articles, including 1294 records from PubMed, 2733 from EMBASE, and 60 from the Cochrane database. After omitting duplicate articles, 3106 articles underwent title and abstract screening for eligibility. Of these, 17 underwent full-text assessment for eligibility. Another 11 articles were excluded, and 6 were eligible for inclusion. Two independent investigators (D.S. and I.H.) agreed to the selection of articles after full-text review. All disagreements were resolved by discussion with a third reviewer (H.J.). Figure 1 depicts the PRISMA flowchart for this study.

### Study characteristics

The characteristics of the included studies are presented in Table 2. Our search yielded four retrospective observational studies and two prospective observational studies. Four studies were conducted on all patient groups, one study was conducted in adult patients, and one study was conducted in pediatric patients. Although the details of each DOR indication were slightly different, the broad inclusion criteria such as persistent hypotension, penetrating injury, evisceration, and amputation were similar among studies. Five studies compared actual mortality with predicted mortality using the trauma score and injury severity score (TRISS), and one study compared mortality using propensity matching. The time to incision was also compared in two studies.

**Table 2:** Characteristics of the six included studies that evaluated the efficacy of direct-to-operating room (DOR) trauma resuscitation

Author	Study setting	Date of recruitment	Intervention group	Study population	DOR indication	Outcome
Wieck et al. [5] (USA)	Prospective study	From 2009 to 2016	82	Pediatric patients	Chest injury Rigid, distended abdomen Evisceration Penetrating injury of the neck, chest, abdomen, and pelvis Traumatic amputation Age-specific hypotension as defined by the ATLS criteria Significant blood loss at the scene or en route Cardiopulmonary arrest due to trauma Physician discretion	Comparison of actual mortality with predicted mortality based on the TRISS Hospital charge
Steele et al. [6] (USA)	Retrospective study	From 1984 to 1995	742	All patients	Cardiac arrest Persistent hypotension (SBP < 100 mmHg) despite administration of intravenous fluid in the field Amputation or uncontrolled external hemorrhage Patients received in transfer from other facilities who had known diagnoses requiring urgent operation	Comparison of actual mortality with predicted mortality based on the TRISS Mean time to incision
Rhodes et al. [7] (USA)	Prospective study	Over 3 years	240	All patients	SBP < 80 mmHg Penetrating torso trauma Multiple long bone fractures Major limb amputation Extensive soft tissue wounds Severe maxilla facial hemorrhage Witnessed arrest	Comparison of actual mortality with predicted mortality based on the TRISS Mean time from leaving the scene to arriving at the OR
Martin et al. [8] (USA)	Retrospective study	From 2000 to 2009	1407	Age > 16 years	Chest injury Rigid, distended abdomen Crush injury to the torso Evisceration Penetrating injury of the neck, chest, abdomen, and pelvis Amputation Profound shock (adult SBP < 80 mmHg, pediatric SBP < 60 mmHg) Massive blood loss at the scene or en route CPR resulting from trauma	Comparison of actual mortality with predicted mortality based on the TRISS Median time to intervention
Johnson et al. [9] (USA)	Retrospective study	From 2012 to 2017	628	All patients	Chest injury Rigid, distended abdomen Crush injury to the torso Evisceration Penetrating injury of the neck, chest, abdomen, and pelvis Amputation Profound shock (adult SBP < 80 mmHg, pediatric SBP < 60 mmHg) Massive blood loss at the scene or en route CPR resulting from trauma Hypothermia (temperature < 31 °C) EMS or flight provider request Ruptured or dissected aortic aneurysm	Comparison of actual mortality with predicted mortality based on the TRISS
Habarth-Morales et al. [10] (USA)	Retrospective study	From 2007 to 2019	133	Age ≥ 15 years (Not referrals from other hospitals)	Penetrating injuries of the neck, chest, abdomen, or pelvis Cardiopulmonary arrest Profound shock Amputation (proximal to the elbow or knee) Open chest or abdominal wound (evisceration) NTDB record from 2013 to 2016 Patients with laparotomy performed within 2 h of ED arrival	Propensity score matching Time to laparotomy incision Blood transfusion requirement ICU length of stay Ventilator day In-hospital mortality

### Efficiency of DOR

The clinical outcomes of the included studies are presented in Table 3. We assessed the results of the five studies that compared the actual mortality related to DOR with the predicted mortality using the TRISS. Wieck et al. conducted a prospective study of pediatric patients and reported no significant difference between the overall and predicted survival rates. However, the actual survival rate of penetrating injuries was higher than the predicted survival rate [5]. Steel et al. [6] conducted a retrospective study that included all age groups and reported that the actual survival rates for overall, blunt, and penetrating injuries were better than the predicted survival rates. Moreover, they also compared the time to incision, revealing a significantly shorter time in the DOR group [6].

**Table 3:** Efficiency outcomes regarding direct-to-operating room (DOR) trauma resuscitation as reported in the six included studies

Author	Mortality	Procedure duration
Wieck et al. [5]	Compared with the predicted survival as calculated by the TRISS model Overall (*n* = 82): 84% observed versus 79% predicted (*p* = 0.4) Penetrating (*n* = 48): 84% observed versus 74% predicted (*p* = 0.002)	Not assessed
Steele et al. [6]	Compared with the predicted survival as calculated by the TRISS model Overall (*n* = 742): 75.3% observed versus 64% predicted (*p* < 0.001) Blunt (n = 255): 63.1% observed versus 52% predicted (*p* < 0.025) Penetrating (*n* = 487): 81.7% observed versus 71% predicted (*p* < 0.001)	Mean time; time to incision (*p* < 0.05) OR resuscitation group requiring major operation within 4 h (*n* = 528): 38.4 ± 1.9 min Non-OR resuscitation group requiring major operation within 4 h (*n* = 1664): 99.4 ± 1.1 min
Rhodes et al. [7]	Compared with the predicted survival as calculated by the TRISS model Overall (*n* = 240): 70% observed versus 62% predicted (p = 0.001) Blunt (*n* = 183): 68% observed versus 57% predicted (*p* = 0.001) AIS-5 head (*n* = 40): 38% observed versus 19% predicted (*p* = 0.02)	Mean time; scene to OR 11.1 min
Martin et al. [8]	Compared with the predicted survival as calculated by the TRISS model Overall (*n* = 1297): 95% observed versus 90% predicted (*p* = 0.01)	Median time; start surgical intervention after arrival 13 min
Johnson et al. [9]	Compared with the predicted survival as calculated by the TRISS model Overall (*n* = 628): 83% observed versus 75% predicted (*p* < 0.01)	Median time; start surgical intervention after arrival Laparotomy: 23 min Damage control: 13 min
Habarth-Morales et al. [10]	EDOR (*n* = 120): 22.5% versus no EDOR (*n* = 120) 15.0% (*p* = 0.14)	Median time; time to incision (*p* < 0.001) EDOR: 25.5 min; IQR, 19–38.5 No EDOR: 40 min; IQR, 28–63

Rhodes et al. [7] prospectively evaluated all patients and reported that the survival rates for overall, blunt, and Abbreviated Injury Scale-5 head injuries were higher than the predicted survival rates and that the mean time from the scene to the OR was 11 min.

Martin et al. conducted a retrospective study with adult patients and reported that the survival rate for overall injuries was better than the predicted survival rate. They also reported a median time to intervention of 13 min [8].

Johnson et al. retrospectively evaluated patients of all ages and reported that the survival rate for overall injuries was higher than the predicted survival rate. They also reported that the median time to surgical intervention was 23 min for laparotomy and 13 min for damage control surgery [9].

Habarth-Morales et al. conducted a retrospective study of adult patients and compared mortality rates using propensity matching. They reported no significant difference in the overall all-cause hospital mortality rates. However, the time to incision in the DOR group was significantly shorter [10].

## Discussion

The importance of adequate prehospital trauma triage for injured patients has been emphasized by previous research [13]. Achieving a shorter time to hemorrhage control following traumatic injury remains a significant challenge in preventing mortality. For rapid hemostasis, the concept of DOR has been proposed long ago, and some institutions have implemented DOR for resuscitating patients with the most severe injuries. In our review, the definition of DOR in most studies was that patients were immediately transported to the trauma OR, bypassing any evaluation in the ER department. The goal of DOR is to minimize any delays to the OR and initiate both resuscitation and surgical interventions [8]. In most studies, the DOR status was determined by the trauma team [5–7, 10]. Martin et al. reported that the best predictors of DOR were mechanism, physiology, and pattern of injury, while EMS suspicion was associated with the least need for DOR [11]. Moreover, most patients assigned to DOR required major surgical interventions, including laparotomy, thoracotomy, craniotomy, and neck exploration, and vascular repair and resuscitation procedures, including intubation, surgical airway, needle decompression, chest tube, central venous access, and massive transfusion [5–7, 9]. The American College of Surgeons certification does not specify requirements for OR locations. Therefore, each trauma center maintains either a rotation OR, a dedicated OR, or an OR located within the ER department [10]. Studies on DOR using EDOR have also been conducted, which were included in our review. There is not much difference between the system or indications of the DOR and EDOR as EDOR refers to ORs located within ER department. Habarth-Morales et al. mentioned that EDOR has several inherent advantages because it provides an ideal location to facilitate diagnosis and intervention, enabling simultaneous resuscitation and hemorrhage control, but the DOR system has same advantages, as mentioned above. In the case of EDOR, patients are triaged directly to the EDOR by either trauma physicians, ED physicians, or trauma nursing staff, according to institutional policies [10]. As DOR or EDOR are terms coined depending on the location, and the underlying central concept is the same, there is no need to interpret the results of studies on DOR and EDOR differently.

Recently, the concept of hybrid OTs has emerged, which has been implemented by many countries. Hybrid OTs allow for simultaneous interventional radiology and operative procedures, thereby shortening the time to definitive treatment. Recent research, including systematic reviews, has investigated hybrid OTs [12, 14–20]. In some studies, the procedural time was reduced by introducing hybrid OTs.

In our review, we identified five studies that compared the actual mortality with the predicted mortality using the TRISS [21]. In four of these studies, the overall survival rate was better than the predicted survival rate. Some studies performed subgroup analyses. Two studies showed that the survival rate for penetrating injuries was better, and one study showed that the survival rate for blunt injuries was better than the predicted survival rate. One study compared mortality rates using propensity matching and found no significant difference in the overall mortality. In addition, some studies assessed the time to the start of surgical intervention. In two studies, the time to the start of surgical intervention was shorter in the DOR group. Although no comparison was made in the three remaining studies, the time to surgical intervention was less than 30 min, suggesting that DOR can reduce the time before institution of hemorrhage control.

Identifying patients who may benefit from DOR remains controversial. Moreover, although there were minute differences in the indications for DOR in each study, they were similar in a broader context. The indications for DOR included (1) profound shock; (2) penetration injuries of the neck, chest, abdomen, and pelvis; (3) cardiopulmonary arrest; (4) amputation; and (5) evisceration. Establishing concrete indications for DOR can help its implementation. To the best of our knowledge, this is the first systematic review to compare the results of DOR in patients with trauma. Our study represents the first attempt to systematically summarize the existing literature using a systematic review to identify comparative studies assessment mortality and the incision time. The results encompass practical aspects that can assist individual hospitals in implementing DOR, given the resource and skill set availability. This review also suggests emerging concepts of hybrid OTs that are associated with the timeliness of the intervention.

Although the results of our systematic review were derived from the best available evidence, there were some limitations. First, the studies reviewed were observational cohort studies; therefore, selection bias may have existed. The use of the Newcastle–Ottawa Scale score did not permit qualitative assessment of the methodological quality of the studies. However, it remains one of the best tools for evaluating non-randomized studies. Second, the included studies did not allow for the quantitative summation of results because most studies compared actual mortality with predicted mortality. In addition, only two studies compared the time to surgical intervention. We suggest that future studies should present their results in broad categories, including the comparison of subgroups that derived clear, practical benefits from DOR and efficiency targets such as 24-h mortality, time to surgical intervention, and amount of blood product involved. Realistically, this can only be achieved in a high-volume center, preferably in a clinical trial setting. Finally, most studies reviewed in our study did not mention the manner in which prehospital interventions, such as airway management, decompression of tension pneumothorax, and blood transfusion or fibrinogen and tranexamic acid use, affected the patients’ outcomes. However, until now, high-level evidence on the relationship between prehospital interventions and patient’s outcomes was lacking; moreover, each country or institute has different emergency medical environments (e.g., the role of EMS, whether medical staff is dispatched to the scene), often limiting interventions that can be performed in the prehospital stage. Moreover, as the scene to OR time for definite bleeding control was as short as 25 min in most studies reviewed in our study, it is difficult to confirm whether interventions at the prehospital stage had a profound effect on the patients’ outcomes. We endeavor to demonstrate this through well-designed follow-up studies in the future. We also suggest that future studies should consider incorporating a multicenter design to compare DOR and traditional practice.

## Conclusions

Based on the results of our systematic review, we conclude that DOR implementation is likely to reduce mortality in patients with trauma and may facilitate rapid intervention in patients with severe shock. Future studies are required for the proper comparison of the efficiency targets, with the possibility of a clinical trial.

### Supplementary Information

**Additional file 1.** The complete search strategy.

## Data Availability

All data generated or analyzed during this study are included in this published article.

## Abbreviations

DOR: Direct-to-operating room resuscitation
OTs: Operating theaters
TRISS: Trauma score and injury severity score
