# Supplementary material for: Efficacy of direct-to-operating room trauma resuscitation: a systematic review
Source: World J Emerg Surg. 2024 Jan 18;19:3. doi: 10.1186/s13017-023-00532-5 (PMC10795202; doi:10.1186/s13017-023-00532-5)
Supplement: Supplementary file 1 — Additional file 1. The complete search strategy. [file 13017_2023_532_MOESM1_ESM.docx]

| **PubMed** |  |
| --- | --- |
| No. | Query |
| #8 | #5 OR #7 |
| #7 | #1 AND #6 AND #3 |
| #6 | "Operating Rooms"[majr] OR "direct to operating room*"[tiab] OR "direct to OR"[tiab] OR "direct transport to operating room*"[tiab] OR "direct transport to OR"[tiab] OR "direct transfer to operating room*"[tiab] OR "direct transfer to OR"[tiab] OR "direct to the operating room*"[tiab] OR "direct to the OR"[tiab] OR "direct transport to the operating room*"[tiab] OR "direct transport to the OR"[tiab] OR "direct transfer to the operating room*"[tiab] OR "direct transfer to the OR"[tiab] OR "operating room resuscitation*"[tiab] OR "place of treatment*"[tiab] OR "treatment place"[tiab] OR "treatment places"[tiab] OR "damage control surger*"[tiab] OR "DCS"[tiab] OR "damage control resuscitation*"[tiab] |
| #5 | #1 AND #2 AND #3 AND #4 |
| #4 | "Mortality"[mesh] OR "Survivors"[mesh] OR "Treatment Outcome"[mesh] OR "mortalit*"[tiab] OR "death rate*"[tiab] OR "fatality rate*"[tiab] OR "CFR"[tiab] OR "survival rate*"[tiab] OR "probability of death*"[tiab] OR "probability of fatalit*"[tiab] OR "probability of surviv*"[tiab] OR "death probabilit*"[tiab] OR "fatality probabilit*"[tiab] OR "survival probabilit*"[tiab] OR ("death*"[ti] OR "fatalit*"[ti] OR "surviv*"[ti] OR "rate"[ti] OR "rates"[ti] OR "rating"[ti] OR "probabilit*"[ti]) OR "survivor*"[tiab] OR "treatment outcome*"[tiab] OR "therapeutic outcome*"[tiab] OR "therapy outcome*"[tiab] OR "outcome*"[ti] OR "effect*"[ti] |
| #3 | "Resuscitation"[mesh] OR "Trauma Centers"[mesh] OR "Time-to-Treatment"[mesh] OR "Time Factors"[mesh] OR "resuscitat*"[tiab] OR "trauma center*"[tiab] OR "trauma unit*"[tiab] OR "emergency center*"[tiab] OR "emergency unit*"[tiab] OR "emergency department*"[tiab] OR "emergency room*"[tiab] OR "time to treatment*"[tiab] OR "door to treatment time*"[tiab] OR "delayed treatment*"[tiab] OR "treatment delay*"[tiab] OR "time factor*"[tiab] OR "time save*"[tiab] OR "time saving*"[tiab] OR "time saved"[tiab] OR "save time"[tiab] OR "saving time"[tiab] OR "saved time"[tiab] |
| #2 | "Operating Rooms"[mesh] OR "direct to operating room*"[tiab] OR "direct to OR"[tiab] OR "direct transport to operating room*"[tiab] OR "direct transport to OR"[tiab] OR "direct transfer to operating room*"[tiab] OR "direct transfer to OR"[tiab] OR "direct to the operating room*"[tiab] OR "direct to the OR"[tiab] OR "direct transport to the operating room*"[tiab] OR "direct transport to the OR"[tiab] OR "direct transfer to the operating room*"[tiab] OR "direct transfer to the OR"[tiab] OR "operating room resuscitation*"[tiab] OR "place of treatment*"[tiab] OR "treatment place"[tiab] OR "treatment places"[tiab] OR "damage control surger*"[tiab] OR "DCS"[tiab] OR "damage control resuscitation*"[tiab] |
| #1 | "Wounds and Injuries"[mesh] OR "Multiple Trauma"[mesh] OR "Shock"[mesh] OR "Hemorrhage"[mesh] OR "trauma*"[tiab] OR "injur*"[tiab] OR "wound*"[tiab] OR "polytrauma*"[tiab] OR "physical insult*"[tiab] OR "hypovolemic shock*"[tiab] OR "circulatory failure*"[tiab] OR "circulatory collapse*"[tiab] OR "bleeding*"[tiab] OR "hemorrhag*"[tiab] OR "haemorrhag*"[tiab] |

| **Cochrane Library** | |
| --- | --- |
| No. | Query |
| #8 | #5 OR #7 |
| #7 | #1 AND #6 AND #3 |
| #6 | [mh "Operating Rooms"[mj]] OR ((direct NEXT/4 operat* NEXT/2 room*):ti,ab,kw) OR direct-to-OR:ti,ab,kw OR direct-to-the-OR:ti,ab,kw OR ((operating-room* NEAR/3 resuscitation*):ti,ab,kw) OR place-of-treatment*:ti,ab,kw OR treatment-place:ti,ab,kw OR treatment-places:ti,ab,kw OR damage-control-surger*:ti,ab,kw OR DCS:ti,ab,kw OR damage-control-resuscitation*:ti,ab,kw |
| #5 | #1 AND #2 AND #3 AND #4 |
| #4 | [mh "Mortality"] OR [mh "Survivors"] OR [mh "Treatment Outcome"] OR mortalit*:ti,ab,kw OR death-rate*:ti,ab,kw OR fatality-rate*:ti,ab,kw OR CFR:ti,ab,kw OR survival-rate*:ti,ab,kw OR probability-of-death*:ti,ab,kw OR probability-of-fatalit*:ti,ab,kw OR probability-of-surviv*:ti,ab,kw OR death-probabilit*:ti,ab,kw OR fatality-probabilit*:ti,ab,kw OR survival-probabilit*:ti,ab,kw OR (death*:ti,kw OR fatalit*:ti,kw OR surviv*:ti,kw OR rate:ti,kw OR rates:ti,kw OR rating:ti,kw OR probabilit*:ti,kw) OR survivor*:ti,ab,kw OR treatment-outcome*:ti,ab,kw OR therapeutic-outcome*:ti,ab,kw OR therapy-outcome*:ti,ab,kw OR outcome*:ti,kw OR effect*:ti,kw |
| #3 | [mh "Resuscitation"] OR [mh "Trauma Centers"] OR [mh "Time-to-Treatment"] OR [mh "Time Factors"] OR resuscitat*:ti,ab,kw OR trauma-center*:ti,ab,kw OR trauma-unit*:ti,ab,kw OR emergency-center*:ti,ab,kw OR emergency-unit*:ti,ab,kw OR emergency-department*:ti,ab,kw OR emergency-room*:ti,ab,kw OR time-to-treatment*:ti,ab,kw OR door-to-treatment-time*:ti,ab,kw OR delayed-treatment*:ti,ab,kw OR treatment-delay*:ti,ab,kw OR time-factor*:ti,ab,kw OR ((sav* NEAR/2 time):ti,ab,kw) |
| #2 | [mh "Operating Rooms"] OR ((direct NEXT/4 operat* NEXT/2 room*):ti,ab,kw) OR direct-to-OR:ti,ab,kw OR direct-to-the-OR:ti,ab,kw OR ((operating-room* NEAR/3 resuscitation*):ti,ab,kw) OR place-of-treatment*:ti,ab,kw OR treatment-place:ti,ab,kw OR treatment-places:ti,ab,kw OR damage-control-surger*:ti,ab,kw OR DCS:ti,ab,kw OR damage-control-resuscitation*:ti,ab,kw |
| #1 | [mh "Wounds and Injuries"] OR [mh "Multiple Trauma"] OR [mh "Shock"] OR [mh "Hemorrhage"] OR trauma*:ti,ab,kw OR injur*:ti,ab,kw OR wound*:ti,ab,kw OR polytrauma*:ti,ab,kw OR physical-insult*:ti,ab,kw OR hypovolemic-shock*:ti,ab,kw OR circulatory-failure*:ti,ab,kw OR circulatory-collapse*:ti,ab,kw OR bleeding*:ti,ab,kw OR hemorrhag*:ti,ab,kw OR haemorrhag*:ti,ab,kw |

| **Embase** |  |
| --- | --- |
| No. | Query |
| #8 | #5 OR #7 |
| #7 | #1 AND #6 AND #3 |
| #6 | ('operating room'/mj OR 'damage control surgery'/exp OR (('direct' NEXT/4 'operat*' NEXT/2 'room*'):ti,ab,kw) OR 'direct to or':ti,ab,kw OR 'direct to the or':ti,ab,kw OR (('operating room*' NEAR/3 'resuscitation*'):ti,ab,kw) OR 'place of treatment*':ti,ab,kw OR 'treatment place':ti,ab,kw OR 'treatment places':ti,ab,kw OR 'damage control surger*':ti,ab,kw OR 'dcs':ti,ab,kw OR 'damage control resuscitation*':ti,ab,kw) |
| #5 | #1 AND #2 AND #3 AND #4 |
| #4 | ('mortality'/exp OR 'survivor'/exp OR 'treatment outcome'/exp OR 'mortalit*':ti,ab,kw OR 'death rate*':ti,ab,kw OR 'fatality rate*':ti,ab,kw OR 'cfr':ti,ab,kw OR 'survival rate*':ti,ab,kw OR 'probability of death*':ti,ab,kw OR 'probability of fatalit*':ti,ab,kw OR 'probability of surviv*':ti,ab,kw OR 'death probabilit*':ti,ab,kw OR 'fatality probabilit*':ti,ab,kw OR 'survival probabilit*':ti,ab,kw OR 'death*':ti,kw OR 'fatalit*':ti,kw OR 'surviv*':ti,kw OR 'rate':ti,kw OR 'rates':ti,kw OR 'rating':ti,kw OR 'probabilit*':ti,kw OR 'survivor*':ti,ab,kw OR 'treatment outcome*':ti,ab,kw OR 'therapeutic outcome*':ti,ab,kw OR 'therapy outcome*':ti,ab,kw OR 'outcome*':ti,kw OR 'effect*':ti,kw) |
| #3 | ('resuscitation'/exp OR 'emergency health service'/exp OR 'time to treatment'/exp OR 'time factor'/exp OR 'resuscitat*':ti,ab,kw OR 'trauma center*':ti,ab,kw OR 'trauma unit*':ti,ab,kw OR 'emergency center*':ti,ab,kw OR 'emergency unit*':ti,ab,kw OR 'emergency department*':ti,ab,kw OR 'emergency room*':ti,ab,kw OR 'time to treatment*':ti,ab,kw OR 'door to treatment time*':ti,ab,kw OR 'delayed treatment*':ti,ab,kw OR 'treatment delay*':ti,ab,kw OR 'time factor*':ti,ab,kw OR (('sav*' NEAR/2 'time'):ti,ab,kw)) |
| #2 | ('operating room'/exp OR 'damage control surgery'/exp OR (('direct' NEXT/4 'operat*' NEXT/2 'room*'):ti,ab,kw) OR 'direct to or':ti,ab,kw OR 'direct to the or':ti,ab,kw OR (('operating room*' NEAR/3 'resuscitation*'):ti,ab,kw) OR 'place of treatment*':ti,ab,kw OR 'treatment place':ti,ab,kw OR 'treatment places':ti,ab,kw OR 'damage control surger*':ti,ab,kw OR 'dcs':ti,ab,kw OR 'damage control resuscitation*':ti,ab,kw) |
| #1 | ('injury'/exp OR 'multiple trauma'/exp OR 'shock'/exp OR 'bleeding'/exp OR 'trauma*':ti,ab,kw OR 'injur*':ti,ab,kw OR 'wound*':ti,ab,kw OR 'polytrauma*':ti,ab,kw OR 'physical insult*':ti,ab,kw OR 'hypovolemic shock*':ti,ab,kw OR 'circulatory failure*':ti,ab,kw OR 'circulatory collapse*':ti,ab,kw OR 'bleeding*':ti,ab,kw OR 'hemorrhag*':ti,ab,kw OR 'haemorrhag*':ti,ab,kw) |
